# Application of the HTA Core Model for complex evaluation of the effectiveness and quality of Radium-223 treatment in patients with metastatic castration resistant prostate cancer

**DOI:** 10.1186/s13561-018-0211-9

**Published:** 2018-10-22

**Authors:** Beata Kiselova Bilekova, Beata Gavurova, Vladimír Rogalewicz

**Affiliations:** 1 Institute of Nuclear and Molecular Medicine, Imaging Diagnostics Department, Rastislavova 785/43, 042 53 Košice, Slovakia; 2 Research and Innovation Centre Bioinformatics, TECHNICOM, Němcovej 5, Kosice, Slovakia; 3 Faculty of Economics, Technical University of Kosice, Němcovej 32, 040 01 Košice, Slovakia; 4 CzechHTA, Faculty of Biomedical Engineering, Czech Technical University in Prague, Nám. Sítná 3105, 272 01 Kladno, Czech Republic

**Keywords:** Metastatic castration-resistant prostate cancer, Health technology assessment, HTA, HTA Core Model, Health technologies and interventions

## Abstract

**Background:**

Health technology assessment (HTA) is currently one of the major challenges in assessing medical innovations and healthcare systems. In Europe, the European Network for Health Technology Assessment (EUnetHTA) has been aspiring to develop and implement standards for international sharing of HTA results and studies. Slovakia and many other EU countries do not have an established HTA system yet. This paper is focused on an exact description of the EUnetHTA Core Model individual domains applied to the process of selecting patients in the terminal stage of prostate cancer for Radium-223 treatment under particular conditions of the Institute of Nuclear and Molecular Medicine (INMM) in Košice, Slovakia.

**Results:**

We produced the first pilot HTA report using the HTA Core Model in Slovakia. The main objective was to collect all relevant information on the particular technology, and provide its summary to the interested stakeholders on one spot. Rather than applying detailed individual items, i.e. assessment elements and assessment element cards, we concentrated on the content of individual domains and tried to fill them with the best country, facility and intervention related data. The dataset consisted of 52 patients that finished the treatment in the period 2015–2017. The patients were carefully selected according to the Radium-223 producer’s criteria. Only 33 patients received the full therapy consisting of six applications; their average survival was 10.5 months from the application of the last dose.

**Conclusions:**

Based on the results of our analyzes, we recommended several changes to the INMM processes and patient follow-up checks during the treatment process in order to make the therapy more effective. The greatest benefit is expected after the implementation of a ^68^Ge/^68^Ga generator in 2018, as the selection of patients suitable for the Radium-223 treatment will improve. We showed that the HTA Core Model can be implemented in Slovakia, even under conditions of no formal HTA support or institutionalization.

## Background

In the context of improving the efficiency of provided health care services, health systems of advanced countries constantly apply new scientific, research and professional information from many disciplines. However, not every new technology or change in diagnostic and/or therapeutic processes results in an improvement in human health. Many experts have explored hospital profitability and looked for appropriate indicators to evaluate the quality and efficiency of the healthcare system for long period (e.g. [3]). In a view of the continuous innovation development and technological progress in health systems, the issue of their financial sustainability becomes one of the most important points. Possibilities how to balance the quality, availability and efficiency of health care are sought [40]. The importance of addressing this issue has increased in relation to the demographic aging processes that take place in many regions nowadays [38]. Health technology assessment (HTA) is currently one of the major challenges in assessing medical innovations and healthcare systems. It is a rapidly evolving multidisciplinary applied-science discipline that evaluates and assesses health technologies and interventions in the context of clinical, ethical, economic, social, legislative, organizational and other parameters, the domains of the rating [5, 13]. It informs decision makers about the benefits, risks and costs of new technologies. Medical technologies in the context of HTA include drugs, diagnostic tests including indicators and reagents, devices, equipment and supplies, medical and surgical procedures, support systems, and organizational and managerial systems used in prevention, screening, diagnosis, treatment and rehabilitation [14]. Health technology assessment is the systematic evaluation of properties, effects or other impacts of health technologies. The main purpose of HTA is to inform policymaking (in the broad sense) in health care [13]. Therefore, HTA must always be based on findings and outputs of research and application of scientific methods.

Genesis of HTA is quite extensive [13, 37, 39]. In the literature, some traces of HTA fragments have been mapped since mid-eighteenth century, but a real flurry of HTA studies appeared only in 1970s and 1980s. In 1972, the United States Congress established the Office for Technology Assessment (not only in health care) [42]. In 1995, the US Congress issued a report describing HTA systems in eight countries [43]. In Europe, the report *Assessing the effects of health technologies* issued by the UK Department of Health in 1991 [41] seems to be crucial in dissemination of HTA methods. In 1990s, regional and international associations were established: INAHTA (1993), ISPOR (1995), and HTAi (2003) as a follower of ISTAHC (1985). In Europe, the HTA activities have been coordinated and developed above all within the European Network for Health Technology Assessment (EUnetHTA) established in 2006. Since 2014, a network of national HTA agencies has been required by Article 15 of the EU Cross-Border Healthcare Directive (2011/24/EU). EUnetHTA has been aspiring to develop and implement standards for international sharing of HTA results and studies. Slovakia, and many other EU countries (Bulgaria, Cyprus, the Czech Republic, Estonia, Greece, Croatia, Malta, Portugal, Romania and Slovenia) do not have an established HTA system yet [18, 21]. It is this lingering fact that motivated our research to apply the HTA Core Model (EUnetHTA, 2016) to a particular medical problem under specific conditions of a healthcare facility in Slovakia, and to point to its wide usability for medical and cost research.

At present, the empirical benefits of HTA are unclear, as there is little evidence on how HTA affects patient health and access to care, and what HTA is causing in hospital budgets and in state budgets [37]. The published analyses are rather theoretical and it is not fully clear, whether the expected benefits have had the expected effects. Some methods used in HTA studies are called into question (e.g. [11, 15, 16, 20, 27, 30]). This situation is even more challenging in Central and Eastern European countries with underdeveloped HTA structures and little experience [20, 31].

The original goal of HTA was to inform the (national) regulator and provide them with further facts useful for policymaking. In the last ten to fifteen years, HTA has been newly used also in strategic decision-making in hospitals [8, 45]; this new application sphere is called hospital-based HTA (HB-HTA), and it has different goals and different tools. HTA Core Model has been designed for the original purpose; however, there is a modified tool called Rapid Relative Effectiveness Assessments (Final Version 4.2) [9], which is more appropriate for HB-HTA. Since the INMM is a unique healthcare facility serving about half of Slovakia, our study can be considered to be drafted for the Slovak national regulator (the Ministry of Health, for evaluation by the Commission). That’s why we decided to apply the full HTA Core Model.

## Methods

The EUnetHTA HTA Core Model is a methodological framework developed by the EUnetHTA European network to standardize HTA reports within the EU in order the HTA studies could be conducted in a structured, uniform format [18, 29]. The HTA Core Model is a registered trademark; its use is subject to a licence [10]. It uses a multidisciplinary approach, and its main objective is to support international collaboration in HTA information production and sharing the results [10, 29, 36]. This model has a structure consisting of nine evaluation domains. Each domain provides tools for tracking the technology description, utilization and impacts from many angles. The individual domains of the HTA Core Model are as follows:

1. Health problems and current technology
2. Description and technical parameters of the technology under review
3. Security
4. Clinical effectiveness
5. Cost and economic evaluation
6. Ethical analysis
7. Organizational aspect
8. Patient and social aspects
9. Legal point of view [10, 23]

This paper is focused on an exact description of the HTA Core Model individual domains applied under particular conditions of the selected healthcare facility – the Institute of Nuclear and Molecular Medicine in Košice, Slovakia (INMM). The subject of our analyses will be diagnostic and therapeutic processes of the selected diagnosis - metastatic castration resistant prostate cancer (mCRPC). Rather than applying detailed individual items (called assessment elements in the HTA Core Model) and assessment element cards, we concentrated on the content of individual domains and tried to fill them with the best country, facility and intervention related data. The objective of the underlying study is not gathering evidence for a decision, whether or not the technology should be reimbursed from the public health insurance, as the insurance companies do it. Its main objective is to collect all relevant information on this technology, and provide its summary to the interested stakeholders (incl. payers, healthcare providers, patients and politicians) on one spot.

The INMM is a state contributory organization - a specialized hospital belonging to the Ministry of Health of the Slovak Republic. It is based in Košice, divided into the following departments: In-patient Department, Imaging Diagnostics Department, Positron Emission Tomography (PET/CT), and Endocrinological Clinic. An INMM subsidiary, the In Vivo Diagnostics Department is located detached in the town of Banská Bystrica.

### Radium-223 in the treatment of metastatic castration resistant prostate carcinoma

Prostate carcinoma is a complicated and interindividually variable tumor. Several stages of its development are recognized from a clinical point of view: localized to the prostate gland, locally advanced and metastatic, that may first be hormonally dependent, later more or less unaffectable by endocrine manipulations, and ending with the death of the affected individual [22]. Life expectation of men with mCRPC ranges from several months to four or more years; mCRPC usually represents the terminal stage of prostate carcinoma. As a standard, the treatment of metastatic hormone-sensitive prostate cancer begins with androgen deprivation therapy, usually followed by chemotherapy after mCRPC has been diagnosed, and the second-line treatment is employed after the chemotherapy possibilities have been exhausted. The following pharmaceutical groups are available in this line: taxane chemotherapy (docetaxel, cabazitaxel), immunotherapy (sipuleucel-T), androgen receptor directed therapies (abiraterone), and radiopharmaceuticals targeted to bone metastases (Radium-223) [1, 2, 35].

These are above all bone metastases that lead to complications such as pain, hypercalcaemia, myelosuppression, spinal cord and spinal nerve roots compression, and, last but not least, pathological fractures. Patients mostly lose weight, feel more tired, and are anaemic and prone to infections. Hence, bone metastases need to be effectively treated since they present a separate disease to a certain extent. The therapeutic options for their treatment are bisphosphonates, denosumab, beta-emitting radionuclide palliative therapy, external radiotherapy, surgery, and, last but not least, new specific radionuclide therapy with an alpha emitter, Radium-223 (^223^Ra) or its dichloride, ^223^RaCl_2_ [32]. Radium-223 is an alpha emitter with a half-life of 11.4 days that is selectively targeted to bone metastases, and, as the only one of the therapeutic options, it has a positive effect on patient survival [4]. The combination of targeted Radium-223 accumulation in the bones, high energy, and short-range alpha particles allows to kill target tumor cells, as it causes deoxyribonucleic acid (DNA) double-strand breaks. It is indicated for the treatment of symptomatic bone metastases in the absence of visceral metastases [28]. In May 2013, Radium-223 received marketing approval by the U.S. Food and Drug Administration (FDA), and in September 2013 an approval in the European Union; it is distributed under the trade name Xofigo® in a form of intravenous injection [19]. Currently, mCRPC is treated with Radium-223 also in Slovakia. In this paper, data from the INMM in Košice and Banská Bystrica are used.

### Selection of suitable patients for treatment with Radium-223

Based on the three-phase double-blind clinical trial of Alpharadin in Symptomatic Prostate Cancer (ALSYMPCA), indication criteria were set for the selection of suitable patients for the treatment with Radium-223 [25, 26]. Alpharadin was the working name of the therapeutic substance containing Radium-223 that is currently registered under the trade name of Xofigo. Men with mCRPC with symptomatic bone metastases (at least two) and no present visceral metastases are indicated for treatment with Radium-223 [4]. Bone metastases are normally displayed by the skeletal scintigraphy examination that should not be older than 6 months at the time of Radium-223 therapy indication. It is not prescribed how to demonstrate the absence of visceral metastases. A suitable method is the whole-body ^18^F-FDG PET/CT scan; in Slovakia, however, this examination is limited due to its lower availability and higher costs [28]. Hematological parameters are also monitored for the selection of suitable patients for the treatment with Radium-223. Before the radiopharmaceutical is first administered, the serum hemoglobin count (HgB) should be ≥100 g/l, the platelet count ≥100 × 10^9^/l, and the absolute neutrophil count ≥1.5 × 10^9^/l. Prior to any subsequent Radium-223 administration, the platelet count should be checked to be ≥50 × 10^9^/l, and the absolute neutrophil count ≥1.0 × 10^9^/l. The hemoglobin count is not checked before further administrations of Radium-223 [44]. It is also recommended that the patients also had lower levels of the prostate-specific antigen (PSA) [17] and the alkaline phosphatase (ALP) [7] in the serum. Taking into account all the facts, the prostate-specific membrane antigen (PSMA) is the best diagnostic method for selecting suitable patients with mCRPC for the treatment with Radium-223; for PET/CT imaging, it can be labeled by the ^68^Ga or ^18^F positron emitter (^68^Ga/^18^F-PSMA-PET/CT), while ^68^Ga is more suitable for better imaging of as many as possible small metastases [4]. In 2018 the INMM plans to purchase a ^68^Ge/^68^Ga generator to produce gallium-68 necessary for ^68^Ga-PMSA-PET/CT metastases imaging in patients with mCRPC.

All indication criteria for treatment with Radium-223 are summarized in Table 1.

**Table 1:** Indication criteria for treatment with Radium-223

prostate cancer stage	metastatic castration resistant prostate cancer
bone metastases	≥ 2 symptomatic bone metastases, skeletal scintigraphy not older than 6 months, other imaging modalities: ^18^F-NaF-PET/CT, ^11^C/^18^F-choline-PET/CT, ^18^F/^68^Ga-PSMA-PET/CT, whole-body MRI
the absence of visceral metastases	lymph nodes ≤3 cm in the short axis are allowed in any location, CT scan, or possibly ^18^F-choline-PET/CT, ^68^Ga-PSMA-PET/CT
symptomatology	analgesic therapy or palliative external radiotherapy due to bone pain
ECOG PS^a^	patients with ECOG 0–2
prediction	expected survival ≥6 months
hematological parameters	hemoglobin ≥100 g/l platelets ≥100 × 10^9^/l neutrophils ≥1.5 × 10^9^/l
contraindications	no contraindications for the Radium-223 therapy are known
warning	increased caution is indicated in patients with inflammatory bowel disease (risk of bowel disease aggravation), with diffuse skeletal metastatic affection, after chemotherapy (increased risk of myelotoxicity - bone marrow damage)

^a^*ECOG PS* Eastern Cooperative Oncology Group Performance Status (assessing the overall patient status in the range of 0–5 points)

Source: Own processing

## Results and discussion

The goal of this chapter is to describe the individual domains of the HTA Core Model applied to the diagnosis of mCRPC under the conditions of the INMM. A qualitative result will be an identification of the strengths and weaknesses of the current diagnostic and therapeutic process that should provide us with specificities allowing a better applicability of HTA under the Slovak conditions in patients with mCRPC. Individual domains of the HTA Core Model are labeled 3.1 to 3.9.

### Health problems and current use of technology (CUR)

Prostate cancer is the second most frequently reported malignant tumor in men in Slovakia. It is also the second most frequent oncological cause of death in men in Slovakia [12]. Thus, it represents one of the main medical problems of the male population, as well as of the Slovak health system. The treatment options vary, depending on the developmental stage of the cancer. The most serious form, in fact the terminal stage, is mCRPC. Patients suitable for the treatment (see above) are referred to a specialized healthcare facility where the therapeutic agent containing Radium-223 is administered to them. In Slovakia, Radium-223 is administered at healthcare facilities in Bratislava, Martin, Nitra, Banská Bystrica and Košice. This paper analyzes patient data from Košice and Banská Bystrica; these two facilities are parts of the INMM. The skeletal scintigraphy is used to localize metastasis sites in bones. If necessary for their selection, the patients can also undergo the ^18^F-PSMA-PET/CT examination. Table 2 shows the amount of treated patients in the INMM in Košice and Banská Bystrica.

**Table 2:** Overview of the number of patients treated in the INMM

Patients	All treated patients	Patients with discontinued treatment	Patients in treatment	Living patients	Dead patients
Košice	45	35	10	16	19
Banska Bystrica	17	17	0	9	8
Together	62	52	10	28	24

Source: Own processing

**Fig. 1:**
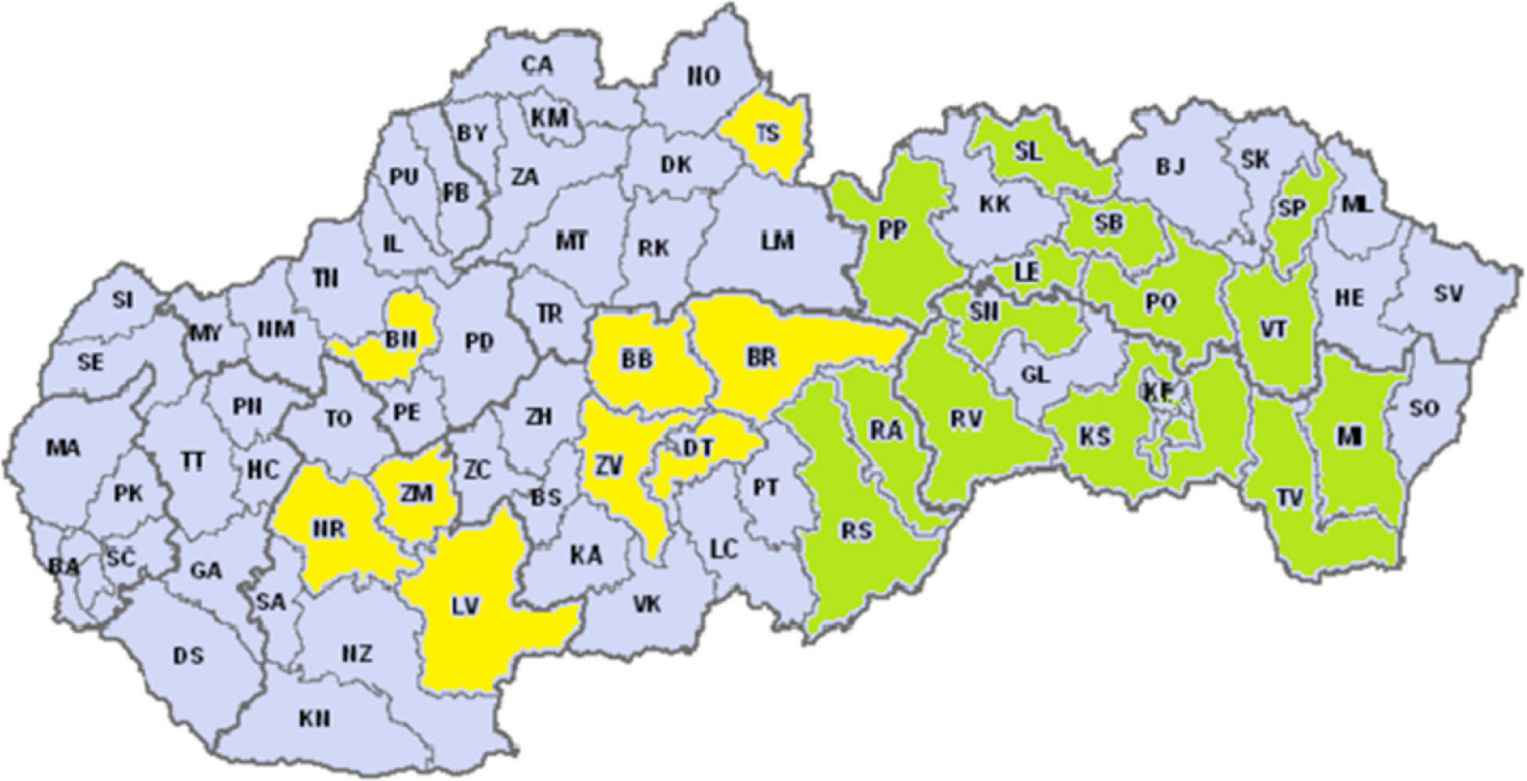
Representation of districts in Radium-223 therapy in the INMM. Explanation to abbreviations: *Districts of Slovakia marked with yellow color:* TS – Tvrdošín, BN - Bánovce nad Bebravou, BB - Banská Bystrica, BR - Brezno, DT - Detva, ZV - Zvolen, NR - Nitra, LV - Levice, ZM - Zlaté Moravce. *Districts of Slovakia marked with green color:* PP - Poprad, SL - Stará Ľubovňa, LE - Levoča, SB - Sabinov, SN - Spišská Nová Ves, RV - Rožňava, RA - Revúca, RS - Rimavská Sobota, KE - Košice, KS - Košice-okolie, PO - Prešov, SP - Stropkov, VT - Vranov nad Topľou, MI - Michalovce, TV - Trebišov

Figure 1 presents a map with marked districts in Slovakia that patients treated with Radium-223 in INMM were from. The districts from which patients were treated in Košice (16 districts) are marked in green, and the districts from which patients were treated in Banská Bystrica (9 districts) are marked in yellow.

The volume of Radium-223 to be administered to a particular patient with mCRPC is calculated on the basis of:

- the patient’s body weight (kg),
- the dose regimen (55 kBq per kg body weight),
- radioactivity concentration of the product (1100 kBq/mL) at the reference date. The reference date is indicated on the injection vial and on the lead pot label,
- the decay correction factor (DK) for the Radium-223 physical decay correction. The correction factor table is provided as a part of the guide to each injection vial.

The amount of radioactivity in the prepared volume must be confirmed by a measurement by a properly calibrated radioactivity meter (dose calibrator). The total volume to be administered to the patient is calculated as follows:

$$ the\ volume\ to\  be\  administered\ (ml)==\frac{body\ weight\ (kg)\times activity\ \left(55\  kBq/ kg\  body\ weight\right)}{DK\  factor\times 1100\  kBq/ ml} $$

Changes due to the daylight saving time are not included in the formula, because changes of 1 h are not considered significant for a radionuclide with a half-life of 11.4 days [44].

### Description and technical characteristics (TEC)

At present, there are three gamma cameras operated in the INMM in Kosice, of which the SPECT/CT is the newest (6 years old) and the other two cameras are older; a public procurement is planned for summer 2018 to replace them by new ones. Since January 2014, the INMM owns a PET/CT that is the only one in Eastern Slovakia. In autumn 2017, a public procurement for a new SPECT/CT took place in Banská Bystrica, where an old camera was also replaced. At present, a pharmaceutical labeled with a metastable isomer of technetium-99 is used for the classical skeletal scintigraphy. For PET/CT examinations, ^18^F is used in the form of fluoro-deoxyglucose (FDG). For patients with mCRPC, ^18^F-PSMA-PET/CT examinations are more appropriate, as they can display metastases in other organs as well. If metastases are too small, they may not show up, and the problem is that even an unsuitable patient can then undergo the Radium-223 therapy. However, this may change as early as 2018, when a public procurement for a ^68^Ge/^68^Ga generator is scheduled.

Within this Core Model domain, staff training should be also evaluated. In the INMM it takes place always when a new technology/device is purchased, and a retraining is organized after sundry updates.

### Safety (SAF)

After an application of Radium-223 to patients with mCRPC, various side effects may occur; however, since the beginning of treatment with this therapeutic agent, skin redness followed by itching at the cannula injection site has been observed in two cases. As this is an outpatient treatment and the patient goes home after the Radium-223 application, the feedback concerning the state after several hours or days is given only during the next application, i.e. a month later. Therefore, it would be practical to introduce questionnaires in the future that patients would get to be filled in at home after each application. Radium-223 is mainly excreted in the stool, approximately 5% being excreted in the urine. The stool excretion can lead to gastrointestinal adverse effects such as nausea, vomiting and diarrhea, which are the most common side effects in the treatment with Radium-223 (≥ 10% of patients). Therefore, it should only be used in patients with Crohn’s disease or ulcerative colitis after a careful consideration of the benefits and risks [6, 44]. According to Sartor [34], the most common adverse effect from the hematopoietic system point of view is thrombocytopenia (≥ 10%), frequent are neutropenia, pancytopenia and leucopenia (≥ 1% to < 10%), less frequent lymphopenia (≥ 0.1% to < 1%). Thrombocytopenia occurred more frequently in patients with superscan identified on the skeleton scintigraphy. In the ALSYMPCA study, the lowest platelet and neutrophil counts occurred after 2–3 weeks after the intravenous administration of Radium-223 injection. Frequently indicated treatment with docetaxel does not significantly affect the safety profile of the radioactive substance. An analysis of the ALSYMPCA study demonstrated that the patients pretreated with docetaxel had a slightly higher hematological toxicity than the chemonaive patients; however the incidence of the grade 3 and/or 4 thrombocytopenia was less than 10% in both groups. Further analyzes shown that Radium-223 can be safely administered both before and after docetaxel, and on the other hand, docetaxel can be administered both before and after the treatment with Radium-223 without any significant effect on hematotoxicity [6].

Within this domain, it is also necessary to investigate the safety of medical staff that come into contact with the patients. Since all patients are radioactive after the administration of Radium-223, adequate protection of workers against ionizing radiation is required. Any of them, whether it is the radiopharmacist preparing the radiopharmaceutical, the physician, the radiology assistant or an orderly, must have a protective lead vest and it is advisable to wear a thyroid shield. All personnel working in the controlled area in the INMM also wear personal dosimeters and staff handling radiopharmaceuticals also finger dosimeters. Both types of dosimeters are being sent for evaluation to the Slovak legal metrology centre, and we can confirm that the maximum permissible dose has not been exceeded in any of the employees yet. However, care must be taken to monitor the staff whether they actually wear protective clothing and instruments, because they often ignore it. Therefore, it would be desirable that a representative for radiation protection carried out spot checks of wearing protective clothing and dosimeters in times of Radium-223 application. The INMM complies with all applicable laws, meets international standards, and has implemented an integrated management system. In January 2018, a certification audit took place in the facility, and the INMM obtained the ISO 9001:2015 (quality management) and ISO 14001:2015 (environment) certificates. As far as Radium-223 is concerned, it is a radioactive substance that the INMM is authorized for. Patient toilets are connected to a collecting system that goes into five delay tanks located underground. They are discharged every 3 months, while the discharge is reported to the Regional Public Health Authority.

When a radioactive substance is applied, contamination of the patient surrounding area may occur since the substance can spray out or run out of the cannula if mishandled. Then, the site is soaked up and covered with white blotting paper, and the representative for radiation protection or his/her deputy measures the site contamination and determines the number of hours or days when the site shell stay covered. The contaminated site is regularly measured and when the sprayed pharmaceutical has fully decayed, the site may be cleaned.

### Clinical effectiveness (EFF)

Treatment with Radium-223 started in the INMM in April 2015. The overview of the number of treated patients is given in Table 3. The average age of the mCRPC patients is 69.7 years with the minimum of 54 years and maximum of 84 years.

**Table 3:** Finished Radium-223 treatment according to the real number of applications

Number of applications	Number of patients treated in Košice	Number of patients treated in Banská Bystrica	Patients together	Percentage [%]
1 application	2	1	3	5.77
2 applications	4	0	4	7.69
3 applications	3	1	4	7.69
4 applications	1	2	3	5.77
5 applications	5	0	5	9.62
6 applications	20	13	33	63.46
Total	35	17	52	100.00

Source: Own processing

Radium-223 is indicated for the treatment of symptomatic bone metastases in the absence of visceral metastases and with node affection ≤3 cm. Totally, six injections are administered to mCRPC patients by slow intravenous injection at 4-week intervals at a dose of 55 kBq/kg patient’s body weight. Hospitalization of the patient is not necessary, the treatment is provided in a hospital outpatient setting [44]. According to the ALSYMPCA study, patients treated with Radium-223 had significantly longer survival as compared to placebo-treated patients (14.9 months vs. 11.3 months). Furthermore, a prolongation of the time to the first symptomatic bone episode (by 5.8 months), a significant improvement in the quality of life, a reduction in the need for external radiotherapy and the use of opioids, a reduction in the risk of spinal cord compression, and a reduction in the need for surgical intervention have been demonstrated. It is recommended that patients completed all 6 treatment cycles if they tolerate it well [25]. An analysis of the ALSYMPCA study indicated that median of overall survival was 17.9 months in patients who received 5–6 injections, and 6.1 months in patients who received 1–4 injections [32]. However, the survival of mCRPC patients was measured from the first Radium-223 dose administered in the referred study, while measuring from the last dose applied is considered more credible. In our work, patient survival and all analyses refer to the date of the last Radium-223 dose administration. Table 3 shows the number of treated patients according to the real number of Radium-223 applications.

The largest share of patients (63.46%) received 6 applications, which is the recommended number. It is desirable that this share is increased at least to 75%.

The average number of months of survival of a patient with mCRPC since the last Radium-223 application is 8.5 months, while the average number of months of survival after the last Radium-223 application of patients that already died is 6 months. The shortest survival time after the last Radium-223 dose was 1 month, while the longest survival was 27 months. Table 4 shows the number of months of survival in all treated patients.

**Table 4:** The survival of all patients since the last application of Radium-223 in months

Number of applications	Patients treated in Kosice	Patients treated in Banská Bystrica	Together
1	6	1.2	4.4
2	7	–	7
3	2.4	1.5	2.2
4	3.5	3.4	3.4
5	6	–	6
6	10.6	10.4	10.5

Source: Own processing

Obviously, the greatest effect on the overall survival since the last application of radium-223 was in those patients with mCRPC that had completed all six cycles of therapy.

Patients' subjective feelings are individual: some patients do not feel the effect of treatment in terms of pain relief, while others feel it already after the first dose. Nevertheless, there are more patients in whom the treatment effect is positive and the pain disappears. For a better evaluation of the Radium-223 effect on patients’ subjective feelings, it would be useful to introduce the above mentioned questionnaires that patients would fill in at home after each application of the therapeutic substance.

The key to the radium-223 treatment success is an appropriate selection of patients [24]. Up to now, it has been performed on the basis of blood test results, skeletal scintigraphy, and 18F-PSMA-PET/CT screening with FDG labeling. It is recommended to indicate the Radium-223 treatment especially in patients with less advanced prostate cancer, who are more likely to undergo the complete treatment and thus benefit more from it. However, the PSA, skeleton scintigraphy, or FDG PET/CT scan are not suitable methods for patient selection or Radium-223 monitoring, as small visceral or other metastases might not be found [4, 7]. Thus, a more precise selection is desirable for selecting suitable patients with mCRPC; a new 68Ge/68Ga generator should help. The 68Ga-PMSA-PET/CT galley imaging is a prostate cancer screening that is characterized by a high specificity and sensitivity, and up to 10% higher diagnostic accuracy as compared to 18F-choline PET/CT. Some studies indicate pathological tissue detection up to 58% at PSA levels 0.2–0.5 ng/ml, or 73% at PSA levels 0.5–1.0 ng/ml, while the reference values are between 0 and 5 ng/ml depending on the laboratory and the analysis method. Because the PET examination allows for body function monitoring, it can help physicians detect deviations in biochemical processes that may indicate the disease before the occurrence of anatomical changes that are detectable by other CT or MRI examinations [2]. Gallium-68 marking is very special and can capture even very small differences, which provides very good conditions for a more precise selection of patients with mCRPC suitable for the Radium-223 treatment. Thanks to this, a premature termination of the treatment can be avoided, which has a positive effect on the amount of costs incurred. The causes of a premature treatment termination in patients treated so far are shown in Table 5.

**Table 5:** Causes of premature termination of the Radium-223 therapy

Patient	Number of Radium-223 injections applied	Number of patients	The cause of a premature treatment termination
1	5 injections	5 patients	brain metastases
2			patient refused the treatment
3			patient refused the treatment
4			progression of the overall condition
5			patient refused the treatment
6	4 injections	3 patients	progression of the overall condition
7			progression of the overall condition
8			neutropenia
9	3 injections	4 patients	progression of the overall condition
10			not identified
11			progression of the overall condition
12			patient stopped walking
13	2 injections	4 patients	anemia and thrombocytopenia
14			brain metastases
15			renal failure
16			progression of the overall condition
17	1 injection	3 patients	brain metastases
18			progression of the overall condition
19			head injury

Source: Own processing

As can be seen from the data in Table 5, the most frequent cause of a premature treatment termination was an overall progression of the disease and the overall condition of the patient, a presence of cerebral metastases, and a worsening of blood parameters. In three cases, patients refused to complete the treatment, but the cause was not examined, which is another reason for introducing the questionnaires and a close collaboration with attending oncologists. It can also be concluded that a better selection of patients with mCRPC for this treatment based on the 68Ga-PMSA-PET/CT scan would reduce the number of patients with less than 5–6 applications. Already the first doses can reduce the spread of metastases, but some patients also experience a temporary deterioration of their condition, while the ultimate effect mean reduced pain and improved quality of life.

### Cost and economic evaluation (ECO)

This domain addresses the cost and economic evaluation of healthcare technologies; a cost-effectiveness calculations (CEA or CUA) are often included. Due to the above-described rather positive situation in the Radium-223 therapy in Slovakia and the main objective of the study, we just calculate the costs of treatment. When treating with Radium-223, in addition to the direct cost of the pharmaceutical, the INMM also bears further costs of consumed materials and staffing. The cost of consumables per an application ranges from EUR 10 to EUR 20, and the cost of the staff is not primarily calculated for the particular treatment, but it is included in total expenses of the in vivo diagnostics cost center. The total price of one Radium-223 package is EUR 5260.13 incl. 10% VAT, which makes EUR 31,560.78 for six doses per patient. Since the treatment with this pharmaceutical started in Slovakia in April 2015, the reimbursement from insurance companies has been decreasing. The point value for one Radium-223 application according to the Reimbursement Decree is 12,000 points, which does not cover the total price for the pharmaceutical; therefore, the supplier makes up for the price in a form of an early payment bonus. Only in this way can the INMM benefit from this treatment, in average EUR 83 per an application. Each insurance company pays a different amount because this treatment is provided in an outpatient department, and thus it is not covered by Diagnosis Related Groups (DRG). Table 6 shows the gradual decrease in the reimbursement for one applied radium-223 dose from insurance companies.

**Table 6:** Gradual decrease in the reimbursements for one applied Radium-223 dose from insurance companies (Dôvera, Všeobecná zdravotná poisťovňa, Union)

month of payment	Dôvera	Všeobecná zdravotná poisťovňa	Union
04/2015 – start of treatment	EUR 4369.54	EUR 5260.18	EUR 4734.17
01/2016	–	EUR 4734.17	–
06/2016	–	EUR 4471.00	–
11/2016	–	EUR 4369.54	–
05/2017	EUR 4267,92	EUR 4347.26	–
11/2017	EUR 3945.14	–	–
02/2018	–	EUR 4267.92	–

Source: Own processing

In December 2017, one person paying cash (i.e. without insurance) began treatment with Radium-223; the health insurance company (Dôvera) set the final price for one applied dose at EUR 3850.00 for him. From the point of view of the public health insurance, the cost of this treatment is high, and thus the most effective selection of appropriate patients with mCRPC is desirable. As mentioned above, 68Ga-PMSA-PET/CT scan seems to be the most appropriate alternative. In 2018 the INMM plans to launch a public procurement to purchase a 68Ge/68Ga generator for production of 68Ga, which is essential for 68Ga-PMSA-PET/CT imaging of metastases that cannot be visualized by conventional scintigraphy or FDG PET/CT. An appropriate selection of patients would also reduce the cost-effectiveness ratio of the therapy, because treatment would not be mostly terminated early, i.e. before the fifth injection application, but a successful completion of the therapy with the sixth dose of Radium-223 would be accomplished. Since the beginning of Radium-223 therapy in the INMM, the treatment was prematurely discontinued in 19 patients out of 62, i.e. 30.6%. After selecting patients based on the 68Ga-PMSA-PET/CT scan, the number of patients terminating their treatment early is assumed to decrease at least to the level of 20%. However, the most important benefit for patients with mCRPC will be their longer survival. The INMM preliminarily surveyed the cost of the 68Ge/68Ga generator including the tools necessary for quality control (Table 7), the costs of consumables for one synthesis (Table 8), and the price of one synthesis (68Ga creation) considering 60 or 100 syntheses per year (Table 9).

**Table 7:** Price of the equipment - generator and quality control tools

Item	Price without VAT	Price incl. 20% VAT
Generator Modular-Lab easy system + Multi Elution Generator Tool	EUR 55382.63	EUR 66459.16
Installation, training, transport	EUR 7647.50	EUR 9177.00
HPLC and TLC with quality control accessories	EUR 54585.33	EUR 65502.39
ALLTOGETHER	EUR 117615.46	EUR 141138.55

HPLC – high-performance liquid chromatography, TLC-thin layer chromatography

Source: Own processing

**Table 8:** Cost of consumables for one synthesis of 68Ga

Consumables	Price without VAT	Price incl. 20% VAT
PSMA	EUR 401.46	EUR 481.75

Source: Own processing

**Table 9:** Price of one synthesis of 68Ga incl. VAT

	Price incl. 20% VAT considering 60 syntheses/year	Price incl. 20% VAT considering 100 syntheses/year
68Ga - PSMA	EUR 1681.00	EUR 1201.30

Source: Own processing

After purchasing the generator, the synthesized 68Ga could be also used to mark the pharmaceuticals needed for examination of e.g. neuroendocrine tumors. Since the closest facility with a 68Ga generator is as far as in Nitra (Izotopcentrum, s.r.o.), the new equipment will find employment in whole Eastern Slovakia and in a part of Central Slovakia.

### Ethical analysis (ETH)

The ethical analysis addresses social and moral standards and values related to the particular technology. It considers the technology itself, and the implications of its inclusion or non-inclusion within the healthcare. From the point of view of medical devices, the ethical questions are formulated in a general way, and they may be specified for individual devices during the process of their operation [10, 33]. The oldest Code of Ethics is the Hippocratic Oath. In addition, the INMM observes that all rules and processes comply with the legislation in force.

Before the treatment of a patient starts, the attending physician-oncologist sends the “Protocol on initiation and control of the treatment of metastatic castration resistant prostate cancer” to the patient’s health insurance company; it includes all due information concerning the patient and his/her treatment up to now. Based on this Protocol, the insurance company either approves or does not approve the Radium-223 therapy. This may result in a social inequality. As this is a very sensitive issue not only for the patient but also for his/her closest family; if the therapy is not approved, both the patients and their family members can consider it as a refusal of physician’s basic duties. However, in this disease, the therapy can be based on results of the 68Ga-PMSA-PET/CT examination that would prove or disprove its suitability. An inappropriate candidate for the Radium-223 treatment would not be reported to the health insurance company.

Since the INMM does not possess a 68Ga generator yet, the patients are selected based on the available methods. However, if the reimbursement of the therapy is refused by their health insurance company, some patients and their relatives may opt for paying for the whole therapy themselves in cash. In this case, it is a question of ethics and morals to allow for the treatment in spite of a bad prognosis, since most people would do anything to give their nearest and dearest a chance for even a shortest prolongation of life. Ethical aspects must always be taken into account also in patients with an impaired ability of decision-making. All ethical issues should be early identified, and efforts should be made to draw standards of how to solve such situations.

### Organizational aspects (ORG)

The organizational aspects domain of the HTA Core Model also focuses on technology delivery, process analysis, resource analysis and management from the perspective of different stakeholders within the health system (EUnetHTA, 2016). Understanding to this domain can reveal further obstacles and problems experienced during implementing medical devices.

From the point of view of the Radium-223 therapy of patients with mCRPC, the right patient selection made by the attending oncologists together with physicians from the INMM is of primary importance. It is preceded by taking a blood sample to determine the required markers, skeletal scintigraphy, and/or PET/CT scan. Table 10 provides a description of patients’ management within the Radium-223 therapy in the INMM. It is very important to monitor the overall health status and blood parameters in order to prevent ordering the radioactive substance that later would not be possible to apply to the patient; such a situation would generate high costs and financial losses for the INMM.

**Table 10:** Patient management within the Radium-223 therapy in the INMM

before starting Radium-223 therapy	patient contacts (mobile phone, e-mail)
	contacts to the referring/collaborating physician, and/or the GP
	verifying the patient’s clinical status and laboratory parameters
	instructions and informed consent (+ 1 copy for the patient)
	the schedule of the Radium-223 application dates and the dates when the results of the differential blood count are to be reported (for the physician and for the patient)
	for the patient - contact to the INMM physician
before ordering the respective Radium-223 dose	differential blood count check - the results to be delivered to the INMM (10 days before the application)
	checking the clinical status of the patient
	ordering the Radium-223 dose for the patient (produced in Norway)
on the day of Radium-223 application	admission of the patient at the INMM reception desk
	taking the patient’s anamnesis and his/her objective examination
	insertion of an intravenous cannula for Radium-223 application
	application of the respective dose of Radium-223, flushing with 0.9% sodium chloride before and after the application
	30 min after the application - measuring of the patient’s dose rate
	discharging the patient from the department approximately 60 min after admission
	for the patient after each application: medical report - Radium-223 treatment protocol, and Statement on radioactive substance application
	for the referring physician after each application: medical report - Radium-223 treatment protocol

Source: Own processing

The patient’s drug security process consists of the following steps:

- 1st step: A nuclear medicine physician, in collaboration with the attending physician (oncologist, urologist), issues an application for a reimbursement approval of a registered medicinal product in the patient’s name for the respective health insurance company. Document: Reimbursement application for a registered medicinal product
- 2nd step: The insurance company decides on the reimbursement of the medicinal product – they will issue a consent to the reimbursement of the Radium-223 registered drug. Document: Opinion on an reimbursement of a registered medicinal product
- 3rd step: Based on the consent of the insurance company to the therapy reimbursement in the patient’s name, the INMM places a binding order to the producer (the Slovak subsidiary of Bayer AG). Document: Hospital’s order (sent by e-mail).
- 4th step: Bayer confirms the order. Document: Order confirmation email from the company.
- 5th step: On the day of Radium-223 delivery, Bayer sends an invoice for the drug to the INMM. Document: Invoice (sent by e-mail)
- 6th step: The insurance company pays for the drug to the hospital (incomplete payment), and the supplier will make up for the drug price.

A confirmation of reimbursement approval issued by the insurance company must be a part of the order. The therapy itself is provided in an outpatient department. Without the consent of the health insurance company, the supplier would not order the drug.

At the end of this domain, it is worth mentioning that a public procurement will take place in the summer 2018 to buy a 68Ge/68Ga generator and the necessary accessories, which will make the selection of patients with mCRPC for the Radium-223 therapy more effective. Nevertheless, a closer cooperation with the attending physicians is desirable in the future, so that the INMM’s competent staff also have complete information on each patient’s treatment, which is not the case yet. It is also necessary for full HTA analyses.

### Patient and social aspects (SOC)

From the patient’s perspective, this domain deals with an assessment of satisfaction with the medical technology. Before any application of the radioactive substance, the patient is briefly interviewed. After any application, the patient is asked how the administration affects his/her psychological and social well-being. Such information can be obtained, in particular, from questionnaires that will be required for the treatment with Radium-223 at the INMM in order to improve the quality of the therapeutic process. For this treatment, the patients create just one homogenous group of men with mCRPC. Some patients and/or their family members are afraid of the radioactive substance, but these are medical radiation doses, and the benefit from the administered substance will outweigh the risk of irradiation. Table 11 compares different dose rates. The data show that the radiation from the Radium-223 administered is almost negligible. Before, during and after the treatment, patients are mainly seeking psychological support from family members and friends. Some patients continue to live in their conventional daily routine, health conditions of some improve, and other patients are getting worse. Within this domain, the possible side effects of treatment, benefits, or harms (injuries, fractures, etc.), which are caused by consequences of the patient’s diagnosis, are also detected. However, a relevant assessment of this domain requires appropriate questions for patients to get credible information for competent staff. Protective regimen should be followed during a week after the Radium-223 administration, especially towards young children and pregnant women, but the presence of older children and adults is possible in this period. However, it is advisable to avoid any physical contact with other people.

**Table 11:** Comparison of different dose exposures

Comparison of different dose exposures in μSv/h	in the distance	
	1 m from the patient	2 m from the patient
3 h after application of Technetium-99 m-labeled pharmaceuticals used in the skeletal scintigraphy	5.4–13.4	not measured
30 min after Radium-223 application	0.376–0.504	0.183–0.205
background in corridors and offices	0.101–0.141	

μSv/h – microsievert/hour

Source: Own processing

The societal influence of the technology cannot be clearly determined, but a prolongation of a higher-quality survival of patients with mCRPC may positively affect death statistics.

### Legal aspects (LEG)

The INMM comply with all respective laws. They operate in compliance with the current legislation and are mindful of patients’ rights. This domain takes into account technology-related legal issues, some of which are directly related to the patient and his/her fundamental rights such as autonomy, informed consent, privacy and confidentiality. According to the guidelines of the HTA Core Model, legal resources were analyzed regarding the treatment of patients at various required levels:

- the European Council level (i.e. the Convention on Human Rights and Biomedicine and the European Convention on Human Rights)
- EU level (the Medical Devices Directive)
- national level
- documentation provided by manufacturers. Informed consent includes the possibility of considering further diagnostic and therapeutic decisions. It informs patients about the risks of the intravenously administered radioactive substance. Patients have time to decide whether to undergo the Radium-223 treatment. This domain should show that the reimbursement of the therapy by insurance companies has been improving, but the results tend to show opposite tendencies in realty. Market regulations for the purchase of medical technologies and the transparency in using public funds are determined by public tendering. The process of public procurement should assure compliance with all legislation in force to achieve an efficient spending of public funds.

## Conclusion

Based on the results of our analyzes, we have come up with several findings pointing to the need for changes in the INMM processes and patient follow-up checks during the treatment process in order to make HTA reports based on the HTA Core Model better and more trustworthy. Since there were only 52 patients treated in the INMM and other 10 still in treatment, it is a small sample for statistics. Over time, patients and their data will be added, helping to better re-evaluate the HTA Core Model application in the hospital. The greatest benefit is expected after an implementation of the ^68^Ge/^68^Ga generator, as the selection of patients with mCRPC suitable for the Radium-223 treatment will improve. The benefits should be a more effective utilization of financial resources and an improved patient quality of life.

On the national level, HTA is not easy to implement. It requires creation of a new agency, and an adaptation of prices and processes of cost reimbursement, which in turn requires a considerable legislative effort. However, the pilot application of the HTA Core Model in the INMM can be done without any major changes (above all legislative ones), which can help (the hospital, the Ministry) to quickly decide on the relative effectiveness of the respective medical technology. A desirable closer collaboration with the attending physicians is intended, and new types of patient questionnaires will be introduced that would be distributed after each application of the radioactive substance and could be taken home for completion. Thus, the INMM will receive a better feedback also from the patients that have already finished the therapy, which should be a step forward in application of the HTA Core Model. As our study shows, the HTA Core Model application under the INMM conditions is challenging, and many data spheres and research areas still need to be specified or put in more precise terms.

## Abbreviations

ALP: Alkaline phosphatase
ALSYMPCA: Alpharadin in Symptomatic Prostate Cancer
Bq: Becquerel
CEA: Cost-effectiveness analysis
CEE: Central and Eastern Europe
CT: Computed tomography
CUA: Cost-utility analysis
CUR: Health problems and current use of technology
DK: Decay correction factor
DNA: Deoxyribonucleic acid
DRG: Diagnosis Related Groups
ECO: Cost and economic evaluation
ECOG PS: Eastern Cooperative Oncology Group Performance Status
EFF: Clinical effectiveness
ETH: Ethical analysis
EU: European Union
EUnetHTA: European Network for Health Technology Assessment
FDA: Food and Drug Administration
FDG: Fluoro-deoxyglucose
Ga: Gallium
Ge: Germanium
HB-HTA: Hospital-based health technology assessment
HgB: Hemoglobin
HPLC: High-performance liquid chromatography
HTA: Health technology assessment
HTAi: Health Technology Assessment International
INMM: Institute of Nuclear and Molecular Medicine
ISO: International Organization for Standardization
LEG: Legal aspects
mCRPC: Metastatic castration resistant prostate cancer
MRI: Magnetic resonance imaging
ORG: Organizational aspects
PET/CT: Positron emission tomography/computed tomography
PSA: Prostate-specific antigen
PSMA: Prostate-specific membrane antigen
Ra: Radium
RaCl_2_: Radium chloride
SAF: Safety
SOC: Patient and social aspects
SPECT/CT: Single-photon emission computed tomography/computed tomography
TEC: Description and technical characteristics
TLC: Thin layer chromatography
VAT: Value-added tax
